# Perceived influence and college students’ diet and physical activity behaviors: an examination of ego-centric social networks

**DOI:** 10.1186/s12889-016-3166-y

**Published:** 2016-06-06

**Authors:** Brook E. Harmon, Melinda Forthofer, Erin O. Bantum, Claudio R. Nigg

**Affiliations:** Social and Behavioral Sciences Division, School of Public Health, University of Memphis, Memphis, TN 38152 USA; Department of Epidemiology and Biostatistics, Arnold School of Public Health, University of South Carolina, Columbia, SC 29208 USA; Cancer Prevention and Control Program, University of Hawaii Cancer Center, Honolulu, HI 96813 USA; Office of Public Health Studies, University of Hawaii, Honolulu, HI 96822 USA

**Keywords:** Obesity, Young adults, Screen time, Nutrition, Exercise

## Abstract

**Background:**

Obesity is partially a social phenomenon, with college students particularly vulnerable to changes in social networks and obesity-related behaviors. Currently, little is known about the structure of social networks among college students and their potential influence on diet and physical activity behaviors. The purpose of the study was to examine social influences impacting college students’ diet and physical activity behaviors, including sources of influence, comparisons between sources’ and students’ behaviors, and associations with meeting diet and physical activity recommendations.

**Methods:**

Data was collected from 40 students attending college in Hawaii. Participants completed diet and physical activity questionnaires and a name generator. Participants rated nominees’ influence on their diet and physical activity behaviors as well as compared nominees' behaviors to their own. Descriptive statistics were used to look at perceptions of influence across network groups. Logistic regression models were used to examine associations between network variables and odds of meeting recommendations.

**Results:**

A total of 325 nominations were made and included: family (*n* = 116), college friends (*n* = 104), high school friends (*n* = 87), and significant others (*n* = 18). Nearly half of participants were not from Hawaii. Significant others of non-Hawaii students were perceived to be the most influential (*M(SD)* = 9(1.07)) and high school friends the least influential (*M(SD)* = 1.31(.42)) network. Overall, perceived influence was highest for diet compared to physical activity, but varied based on comparisons with nominees’ behaviors. Significant others were most often perceived has having similar (44 %) or worse (39 %) eating behaviors than participants, and those with similar eating behaviors were perceived as most influential (*M(SD)* = 9.25(1.04)). Few associations were seen between network variables and odds of meeting recommendations.

**Conclusions:**

Among the groups nominated, high school friends were perceived as least influential, especially among students who moved a long distance for college. Intervention strategies addressing perceived norms and using peer leaders may help promote physical activity among college students, while diet interventions may need to involve significant others in order to be successful. Testing of these types of intervention strategies and continued examination of social networks and their influences on diet and physical activity behaviors are needed.

## Background

Obesity has been a major concern in the United States with high prevalence rates and little improvement in the past 10 years [1]. In 2012, 40 % of college-aged adults (ages 18–24) were classified as either overweight or obese [2], which marks the start of a trend towards larger percentages of overweight and obesity among adults in later stages of life [1, 2]. This trend suggests the need for interventions aimed at obesity-related behaviors among college-aged adults as a strategy for reducing rates and long-term health problems among adults [2, 3]. Recent studies have found obesity to be, in part, a social phenomenon and suggest that tapping into social networks is necessary for successful weight loss interventions [4, 5].

In 2012, 41 % of young adults between the ages of 18 and 25 reported being enrolled in either a 2-year or 4-year college [6]. Transitioning away from home and attending college brings the potential for changes in lifestyle behaviors [7, 8] and support systems [9, 10]. Moreover, college students in general have low physical activity rates and poor dietary habits [11, 12]. Although a number of studies have examined social influences on adolescent diet and physical activity behaviors [5, 13, 14], less work has considered such pathways among college students.

Some have argued the dynamic between parent, family, and peer support and influence shifts, with peers becoming a greater source of support and influence as young adults transition away from home [9, 10]. However, other research indicates there may be continuity in actual support, with changes occurring in how college students perceive support from parents compared to peers [15]. In addition, perceptions and actual influence changes may be behavior-specific [16, 17], and the behaviors of those providing support may be as important as the source of the support. Research suggests how an individual perceives their support system and environment, sometimes called social, perceived, or descriptive norms [18, 19], has an influence on behavior and health regardless of whether that perception is true [19–21]. To date, much of the literature has focused on the diet and physical activity behaviors of adolescents and children [22–25]. In contrast, very little is known about the sources of influence on college students’ diet and physical activity behaviors and the influence of perceived norms on these behaviors [17].

This study aimed to examine college students’ social networks related to diet and physical activity, perceptions of network members’ influence on behaviors, perceptions of how the behaviors of network members compared to students’ own behaviors, and associations between network variables and college students meeting diet and physical activity recommendations.

## Methods

Forty college students recruited from three campuses within the University of Hawaii system, one four-year campus and two two-year campuses, completed questionnaires and participated in focus groups aimed at understanding the diet and physical activity behaviors of college students. Eligible participants were 18 years of age or older and enrolled in the University of Hawaii system at the time of the study. All recruitment protocols and materials were approved by the University of Hawaii Manoa Internal Review Board before recruitment began, and all participants provided written informed consent. Recruitment efforts occurred on all campuses between September 2013 and January 2014 using a combination of posted flyers and recruitment tables. Participants were compensated for their time with a $15 gift card.

Participants completed a total of eight questionnaires related to diet and physical activity behavior change. The current analysis included data from the demographic questionnaire, the National Cancer Institute Fruit and Vegetable Screener [26], the National Cancer Institute Percentage Energy from Fat Screener [27], questions on time spent in physical and sedentary activities from Project EAT, a longitudinal study of health behaviors from adolescents into young adulthood [28–30], and a name generator tailored to the study’s aims. The name generator [31] asked participants to nominate “people that are currently part of the groups below and who you consider important to you” the groups included: “your family,” “friends from your time in college,” “friends from your time in high school,” and “significant other.” Participants could nominate up to five people in each network and were asked to record each nominee’s gender, length of time known, and number of times the nominee was seen in the past seven days. This study was ego-centric in that it focused on the networks from the participants’ perspectives and did not include data provided by each nominee [31].

Participants were asked how influential each nominee was on the participant’s eating and physical activity behaviors (1 = not at all to 10 = greatly influences), and how the nominee’s eating and physical activity level compared to the participant (better, same as, or worse). The conceptualization and measurement of perceived norms and their influence on behavior has varied in the literature. Our question on the influence of nominees was adapted from questions on social influences used in Project EAT and a study of perceived norms among young adults [17, 32, 33]. Literature in which young adults were asked to compare nominee’s eating and physical activity levels to their own was not found. Therefore, our questions were adapted from studies where adolescents and children were asked to report on their behaviors and report on the behaviors of their peers [19–21].

Unique identification numbers were assigned to participants and nominees to track if individuals were nominated in multiple networks. Only two participants listed the same friend in both their high school and college friend networks, and these nominees were kept in both networks. Data were aggregated to provide nomination counts in each network and means for continuous variables (i.e. time known, days seen, perceived influence). Means and standard deviations were weighted to reflect the total number of nominees in each network. Data were also aggregated by the three comparison groups (better, same as, worse) for eating and physical activity, and means for perceived influence were calculated. As these data were not normally distributed, the non-parametric Kruskal-Wallis test was used to examine means across the three comparison groups for each behavior.

Nearly 43 % of participants were not from Hawaii, which provided a unique opportunity to examine how separation from networks established before college might impact network make up and influence on current behaviors. Networks established during adolescence and childhood have been found to be influential on diet and physical activity behaviors; however, less is known about the persistence of this influence as young adults leave home and connect with a significant other [24, 33], especially when the move is geographically far. Therefore, network nominations and perceptions of influence are presented for the entire dataset as well as stratified by whether participants were from Hawaii or not from Hawaii. As data were not normally distributed, the non-parametric Mann–Whitney *U* test was used to examine differences between Hawaii and non-Hawaii students for mean nominations, frequency of contact, influence on eating, and influence on physical activity.

Logistic regression models were run to examine associations between network variables and the odds of meeting recommendations related to time spent in moderate-to-vigorous physical activity (MVPA), defined as 30 min or more per day spent in MVPA [34], and screen time (i.e., watching TV or using a computer outside of homework), defined as less than two hours per day [35]. Dietary intake was skewed with most participants meeting the recommendation for fat intake (less than 35 % of daily caloric intake from fat) [36] and few participants meeting the recommendation for fruit and vegetable intake (at least five cups per day of fruits and vegetables) [37]. Therefore, cut points were created at the 33^rd^ and 66^th^ percentiles of the distribution for both percentage of calories from fat (fat intake) as well as servings of fruits and vegetables. Logistic regression models were run to examine associations between network variables and the odds of being in the top tertile for each dietary behavior. For fat intake, the top tertile included participants with 33.7 % of calories or more coming from fat, and fruit and vegetable intake’s top tertile included participants eating 3.2 servings or more per day.

In each logistic regression model, control variables were entered into the first block. These included the categorical variables sex (male or not) and ethnicity (Asian American or not) as well as continuous variables: age, hours a day of MVPA (in the screen time, fat intake, and fruit and vegetable models), hours a day of computer and TV time (in the MVPA, fat intake, and fruit and vegetable models), percentage of daily calories from fat (in the MVPA, screen time, and fruit and vegetable models), servings of fruits and vegetables per day (in the MVPA, screen time, and fat intake models). The socio-demographic variables controlled for in this analysis are commonly controlled for in diet and physical activity behavior analyses [5, 25, 33]. Given the body of literature indicating associations between diet, physical activity, and sedentary behaviors [30, 38, 39], we controlled for these variables in our analyses.

Network variables were included in the second block and included total network nominations (summed across all four networks) as well as mean influence on physical activity (summed across all four networks) for MVPA and screen time models and mean influence on eating (summed across all four networks) for the dietary fat and fruit and vegetable intake models. Due to the ego-centric nature of our dataset, we were limited in the network variables that could be considered; however, network size and influence of nominees have been shown to be important variables to examine [17, 19, 21, 25, 33]. Odds ratios with 95 % confidence intervals were examined for each variable. The *χ*^2^ and Nagelkerke R^2^ values for each block and the overall models were examined to provide an assessment of the amount of additional variance explained by the network variables. All data analysis was conducted using SPSS software (v. 22, IBM Corp).

## Results

Participants in the study were primarily female (65 %) and Asian American (33 %) or of mixed ethnicity (30 %). Among participants who identified themselves as mixed ethnicity, Asian American, white, and Native Hawaiian had the highest frequencies. Approximately half of participants reported living with their parents (55 %). Most met recommendations related to diet and physical activity except for intake of fruits and vegetables (Table 1).

**Table 1:** Demographics of college students who completed social network questionnaires (*n* = 40)^a^

Age (y), Mean (SD)	25.4 (7.9)
Gender	
Female	26 (65)
Male	14 (35)
Ethnicity	
Asian American	13 (32.5)
Mixed Ethnicity	12 (30)
White	8 (20)
Other (Native Hawaiian, Latino, African American)	7 (17.5)
Home State	
Hawaii	23 (57.5)
Not Hawaii	17 (42.5)
Lived in the Past Year	
Parent’s Home	22 (55)
Independent	10 (25)
Residence Hall	4 (10)
Other	4 (10)
Diet and Physical Activity Behaviors, Mean (SD)	
Fruit and Vegetable Intake (cups/day)	2.9 (2.6)
Fat Intake (% of daily energy)	31.0 (6)
Moderate-Vigorous Physical Activity (hours/week)	3.6 (3.4)
Screen Time (hours/week)	10.1 (5.3)
Meets Recommendations, % yes	
Fruit and Vegetable Intake (5 cups/day)	5 (12.5)
Fat Intake (20–35 % of daily energy)	29 (72.5)
Moderate-Vigorous Physical Activity (≥30 min/day)	22 (55)
Screen Time (≤2 hours/day)	30 (75)

^a^Data are presented as *N* (%) unless otherwise noted

The 40 students nominated 325 people with the following types of relationships: family (*n* = 116), college friends (*n* = 104), high school friends (*n* = 87), and significant others (*n* = 18) (Table 2). All network nominations ranged from zero to five except significant other, which ranged from zero to one. Nominations in each network did not statistically differ between students from Hawaii and those not from Hawaii with family nominees approximately 36 % of nominations, college friends 32 %, high school friends 27 %, and significant others 6 %. Hawaii students and non-Hawaii students statistically differed in the number of times they reported seeing high school friends (*M(SD)* = 0.92(1.46) compared to 0(0), *p* < .001) as well as family (*M(SD)* = 5.26(2.66) compared to 1.36(1.83), *p* < .001) in the past week (Table 2). The number of times college friends and significant others were seen did not statistically differ between Hawaii and non-Hawaii students, and significant others were reported as being seen most often across networks (*M*_overall_(*SD)* = 6.83(2.01)).

**Table 2:** Characteristics of network nominations for all students, students from Hawaii, and Non-Hawaii students

Networks	Total nominations (*n* = 325) *N* (%)	Nominations by Hawaii students^a^ (*n* = 187) *N* (%)	Nominations by Non-Hawaii students (*n* = 138) *N* (%)	Days seen in past week Mean (SD)	Times seen in past week Hawaii students^a^ Mean (SD)	Times seen in past week Non-Hawaii students Mean (SD)
Family	116 (35.7)	66 (35.3)	50 (36.2)	3.58 (3.03)	5.26 (2.66)*	1.36 (1.83)
College Friends	104 (32.0)	59 (31.6)	45 (32.6)	2.91 (1.77)	2.58 (1.19)	3.36 (2.26)
High School Friends	87 (26.8)	52 (27.8)	35 (25.4)	0.55 (1.21)	0.92 (1.46)*	0 (0)
Significant Others	18 (5.5)	10 (5.3)	8 (5.8)	6.83 (2.01)	6.40 (2.46)	7.38 (1.19)

^a^Mann–Whitney *U* test of mean differences between Hawaii and non-Hawaii Students, two-tailed

*Means were statistically different between Hawaii and non-Hawaii students, *p* < 0.001

**Fig. 1:**
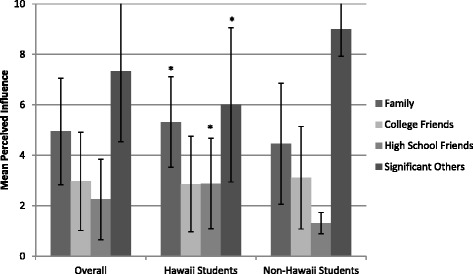
Mean Perceived Influence of Network Nominees on Eating Behaviors of Participants with Standard Deviations. *Networks where a statistically significant difference was seen between Hawaii and non-Hawaii students, *p* ≤ 0.05

**Fig. 2:**
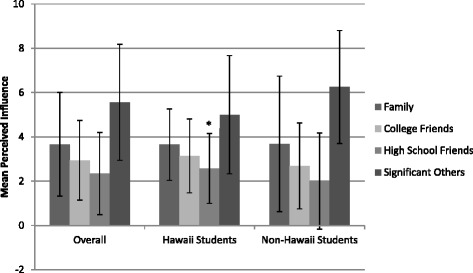
Mean Perceived Influence of Network Nominees on Physical Activity Behaviors of Participants with Standard Deviations. *Networks where a statistically significant difference was seen between Hawaii and non-Hawaii students, *p* ≤ 0.05)

When perceived influence on eating was examined (Fig. 1), significant others and family members were noted as having the highest influence (*M(SD)* = 7.33(2.79) and 4.95(2.11), respectively). The perceived influence of significant others was highest (*M(SD)* = 9(1.07)) and the perceived influence of high school friends lowest (*M(SD)* = 1.31(.42)) among non-Hawaii students. Perceived influence on eating for these two networks, along with family, statistically differed from Hawaii students. (*p* = .03, <.001, .05, respectively). Similar trends were seen with perceived influence on physical activity (Fig. 2), although only perceived influence of high school friends was statistically different between Hawaii and non-Hawaii students (*M(SD)* = 2.58(1.58) compared to 2(2.17), *p* < .001). In addition, perceived influence across networks was lower for physical activity compared to eating.

Tables 3 and 4 present data on the alignment of participants with network nominees they perceived as having similar, better, or worse behaviors than their own. Regarding eating behaviors (Table 3), participants believed college and high school friends had better eating habits (42 % and 40 %, respectively) and that family members had worse eating habits (39 %) than themselves. Significant others were more often categorized as eating the same as (44 %) or worse (39 %) than the participant. When perceived influence on eating was assessed across categories, a significant difference was seen in the influence of family members and significant others. For family nominees, a gradient was seen in which perceived influence was inversely associated with ratings of better eating behaviors. Those perceived as eating worse than participants had the lowest influence (*M(SD)* = 3.93(2.34)) and those perceived as eating better had the highest influence (*M(SD)* = 6.44(2.56)). In contrast, significant others rated as eating the same as participants had the highest perceived influence (*M(SD)* = 9.25(1.04)). When frequency of contact was examined, perceived influence was only associated for significant others (data not shown). Frequency of contact with significant others who were perceived to eat better was considerably lower (*M(SD)* = 3.67(3.22), *p* = .01) than frequency of contact for those perceived to eat the same (*M(SD)* = 7.13(1.13)) or worse (*M(SD)* = 7.86(0.38)).

**Table 3:** Perceived influence on eating by perceived comparison category for each network

Networks	Comparison categories	Comparison to Nominee’s eating behaviors, *N* (%)^a^	Influence on eating, Mean (SD)	*χ* ^2^ (*p*-value)^b^
Family	Better	41 (35.3)	6.44 (2.56)	23.27 (<0.001)
	Same as	30 (25.9)	4.43 (2.50)	
	Worse	45 (38.8)	3.93 (2.34)	
College Friends	Better	44 (42.3)	3.11 (1.86)	2.93 (0.23)
	Same as	38 (36.5)	3.03 (2.24)	
	Worse	22 (21.2)	2.59 (2.49)	
High School Friends	Better	35 (40.2)	2.40 (1.74)	1.61 (0.45)
	Same as	29 (33.3)	2.55 (2.10)	
	Worse	23 (26.4)	1.65 (0.82)	
Significant Others	Better	3 (16.7)	5.67 (2.08)	7.71 (0.02)
	Same as	8 (44.4)	9.25 (1.04)	
	Worse	7 (38.9)	5.86 (3.24)	

^a^Percentages add up to 100 within each cell

^b^Kruskal-Wallis test of mean influence on eating across three comparison categories, two-tailed tests for significance

**Table 4:** Perceived influence on physical activity by perceived comparison category for each network

Networks	Comparison categories	Comparison to Nominee’s physical activity, *N* (%)^a^	Influence on physical activity, Mean (SD)	*χ* ^2^ (*p*-value)^b^
Family	Better	46 (39.7)	4.87 (2.74)	19.33 (<0.001)
	Same as	21 (18.1)	3.86 (2.67)	
	Worse	49 (42.2)	2.45 (2.08)	
College Friends	Better	48 (46.2)	3.77 (1.90)	17.48 (<0.001)
	Same as	32 (30.8)	2.34 (1.82)	
	Worse	24 (23.1)	2.08 (1.73)	
High School Friends	Better	33 (37.9)	2.73 (2.20)	2.67 (0.26)
	Same as	23 (26.4)	2.52 (2.01)	
	Worse	31 (35.6)	1.81 (1.60)	
Significant Others	Better	5 (27.8)	4.20 (2.17)	3.15 (0.21)
	Same as	8 (44.4)	6.63 (2.39)	
	Worse	5 (27.8)	5.20 (3.11)	

^a^Percentages add up to 100 within each cell

^b^Kruskal-Wallis test of mean influence on physical activity across three comparison categories, two-tailed tests for significance

With physical activity (Table 4), participants reported more college and high school friends were better (46 % and 38 %, respectively) than they at being active, more family members (42 %) were reported as being worse, and more significant others were reported as being the same as (44 %) participants. When perceived influence on physical activity was assessed across comparison categories, a significant difference was seen in the influence of family members and college friends. A gradient was seen in family and college friend networks with those perceived as being less active having the lowest influence (*M(SD)* = 2.45(2.08) and 2.08(1.73), respectively) and those perceived as more active having the highest influence (*M(SD)* = 4.87(2.74) and 3.77(1.9), respectively). Perceived influence was not associated with frequency of contact for physical activity (data not shown).

Neither control nor network variables significantly predicted meeting MVPA recommendations or being in the top third for fruit and vegetable intake (Table 5). More network nominations were associated with a higher odds of meeting screen time recommendations (OR = 1.29, 95 % CI = 1.00, 1.65) and adding network variables helped to explain an additional 13 % of the variance in that model. With fat intake, older age was the only significant association and it was associated with higher odds of being in the top tertile (OR = 1.14, 95 % CI: 1.00, 1.30).

**Table 5:** Odds ratios for college students having healthier diet and physical activity behaviors (*n* = 40)

Model variables	*X* ^2^ (df)	*p*-value	R^2, d^	Odds Ratio	95 % Confidence interval	
					Upper	Lower
MVPA (≥30 minutes of PA/day)^a^	9.61 (8)	0.29	0.29			
Control Variables	8.68 (6)	0.19	0.26			
Sex				1.43	0.30	6.88
Age				1.02	0.92	1.15
Ethnicity				0.27	0.05	1.58
Calories from Fat				0.97	0.83	1.13
Screen Time				0.66	0.23	1.89
Fruit and Vegetable Intake				1.43	0.87	2.34
Network Variables	0.94 (2)	0.63	0.03			
Network Nominations				1.09	0.90	1.31
Perceived Influence on PA				1.03	0.69	1.55
Screen Time (≤2 hours screen time/day)^b^	9.86 (7)	0.20	0.32			
Control Variables	4.63 (6)	0.59	0.16			
Sex				0.41	0.07	2.33
Age				1.08	0.94	1.24
Ethnicity				0.29	0.03	2.42
MVPA Time				1.07	0.11	10.28
Calories from Fat				0.84	0.68	1.05
Fruit and Vegetable Intake				1.23	0.73	2.08
Network Variables	4.05 (2)	0.05	0.13			
Network Nominations				1.29*	1.00	1.65
Perceived Influence on PA				0.70	0.40	1.21
Calories from Fat (20-35 % of calories/day)^c^	9.97 (7)	0.19	0.32			
Control Variables	5.92 (5)	0.31	0.20			
Sex				0.58	0.10	3.45
Age				1.14*	1.00	1.30
Ethnicity						
MVPA Time				0.33	0.05	2.27
Screen Time				0.55	0.16	1.91
Fruit and Vegetable Intake				1.24	0.90	1.70
Network Variables	4.05 (2)	0.13	0.12			
Network Nominations				1.22	0.97	1.52
Perceived Influence on Eating				1.30	0.81	2.09
Fruit and Vegetable Intake	5.90 (8)	0.66	0.20			
Control Variables	4.89(6)	0.56	0.17			
Sex				0.97	0.20	4.75
Age				0.93	0.82	1.05
Ethnicity				0.36	0.04	3.01
MVPA Time				2.15	0.41	11.31
Screen Time				0.97	0.33	2.82
Calories from Fat				1.02	0.88	1.19
Network Variables	1.01(2)	0.60	0.03			
Network Nominations				0.95	0.77	1.16
Perceived Influence on Eating				0.78	0.47	1.29

^a^Sex (Male = ref), Age, ethnicity (Asian Americans = ref), percentage of daily calories from fat, computer and TV time (hours/day), total network nominations, mean influence of networks on physical activity

^b^Sex (Male = ref), Age, ethnicity (Asian Americans = ref), percentage of daily calories from fat, moderate-to-vigorous physical activity (hours/day), total network nominations, mean influence of networks on eating

^c^Sex (Male = ref), Age, computer and TV time (hours/day), moderate-to-vigorous physical activity (hours/day), total network nominations, mean influence of networks on eating

^d^Nagelkerke R^2^, two-tailed tests for significance

*p ≤ .05

## Discussion

College is a critical point and location where obesity prevention programs could change the trajectory of college-aged adults gaining weight and increasing their risk of obesity-related health problems later in life. While research has shown diet, physical activity, and obesity to be influenced by social networks, little is known about the networks of college students. Understanding the composition of these networks is important given the potential for shifts to occur in source of support and the influence of these networks as students move away from home and develop new ties. Examining networks and their influence on diet and physical activity behaviors may also provide avenues for future obesity-related interventions.

Findings from this study indicate significant others, family, and college friends are potentially influential for both diet and physical activity behaviors. The level of influence was dependent on the behavior and on perceptions of the nominees’ behaviors. In addition, having a higher number of network nominations was associated with greater odds of meeting screen time recommendations. These findings provide potential strategies for future diet and physical activity interventions aimed at college students.

College enrollment is expected to grow by 13 % over the next six years [40], and the distance traveled for college may have important implications for the social support students receive from old and new family and friend networks. Stratification by whether students were from Hawaii or not showed similar percentages of nominations in the five network groups assessed even though students not originally from Hawaii were significantly less likely to have seen family and high school friends in the past week compared to students from Hawaii. While we did not ask participants to distinguish between seeing nominees face-to-face or via technology (e.g., Skype, Facetime), emerging technology may help young adults remain connected to networks at home [41]. However, our finding that in general participants reported seeing significant others most often and high school friends least often suggests shifts are occurring in college students’ social networks that may have important implications for behavior change.

While students from Hawaii perceived significant others as being most influential on their eating behaviors, family was a close second, and college and high school friends were seen as having similar levels of influence. In contrast, students not originally from Hawaii perceived significant others as more influential than any of the other networks, with a mean perceived influence that was statistically higher than that of students from Hawaii. Although students who were not from Hawaii reported not seeing family often, family still remained the second highest group in perceived influence, with high school friends perceived as having the lowest level of influence of any network. The literature on adolescence and peer support stresses the influence of school friends [5, 24]; however, once individuals enter college the combination of distance from high school friends and the emergence of relationships with a significant other may counteract the influence of friends from earlier points in life. Friends formed in college were an area of influence in this study, but overall family remained more influential than friends. These findings reinforce the need for interventions aimed at improving the diet and physical activity behaviors of parents as their influence can last well into their children’s adult lives [7, 42]. Similar patterns of perceived influence were seen with physical activity, but participants reported network members having a higher influence overall on diet than on physical activity. The social context of many eating occasions [43] may partially explain this difference.

One often cited feature of social networks is homophily, or the tendency for individuals to affiliate with others who are like themselves [31]. In our study, significant others was the only network where participants rated most members as having similar levels of behavior. College friends were perceived to have better physical activity behaviors, which was significantly associated with higher perceived influence. This finding suggests that while physical activity behaviors among college friends may not exhibit a strong tendency toward homophily, they do provide a potential area for intervention. Strategies based on diffusion of innovations theory and aimed at spreading physical activity behavior change through a network of college friends may be effective, as it appears these networks include “innovators” of this behavior [44–46]. However, perceptions of influence were lower for physical activity, compared to diet, suggesting more research is needed in this area to understand social influences.

While it was not significant for every network, a gradient was seen in influence with the highest influence associated with nominees perceived as having better behaviors. This gradient was seen across networks for both behaviors. The only network where this did not occur was with significant others. For both diet and physical activity, significant others who were perceived as having the same level of behavior were perceived as the most influential. This association was significant for dietary behaviors suggesting the need to build interventions for young adults that include significant others, especially when the interventions are aimed at diet. This strategy is supported by previous research, which found associations between increased fruit and vegetable intake and physical activity among college students whose significant other had positive healthy attitudes [16, 33].

Using logistic regression models, we found those students who made more nominations, across all networks, were more likely to meet screen time recommendations. Reducing screen time, independent of increasing physical activity levels, has proven to be important in reducing obesity and chronic disease [47]. Our finding suggests one avenue to reduce college students’ screen time may be helping them build social connections. No studies were found examining the role of social networks and reduced screen time or sedentary time, suggesting the need for additional research in this area.

The study’s sample size was small, data were cross-sectional in nature, and data collection occurred only in Hawaii. These limitations reduce the generalizability of our findings and potentially contributed to the lack of associations seen in our logistic regression models. We also assessed perceived versus actual behaviors of nominees. Future work should measure both to clarify the relationship between the behaviors of those in an individual’s network and their influence.

Participants in this study had a mean age of 25.4, making some older than most undergraduates; however, reliance on data from traditional 4-year college students to describe the college experience has been criticized [48]. In addition, the number of participants who came from outside of Hawaii allowed us to examine differences in networks and network influence among those who remain close to home for college compared to those who move away. Despite its limitations, this study provides unique and important insight into the networks of young adults and avenues to pursue with future studies.

## Conclusions

This study provides an initial look at social networks and their influence on the diet and physical activity behaviors of college students. Study results indicate these networks may still be grounded in family, but are also shifting to incorporate college friends and significant others. Distance from home, the type of behavior, and perceptions of nominees’ behaviors all impact how influential network groups are on college student’s personal diet and physical activity behaviors. Interventions need to account for network structures and perceptions of influence in order to effectively impact college students’ diet and physical activity behaviors. In addition, continued examination of social networks and their influences on diet and physical activity behaviors is needed.

## Abbreviations

M, Mean; MVPA, Moderate-to-vigorous physical activity; N/n, Number; SD, Standard deviation; TV, Television; y, Years.
